# Supplementary material for: mTOR-dependent phosphorylation controls TFEB nuclear export
Source: Nat Commun. 2018 Aug 17;9:3312. doi: 10.1038/s41467-018-05862-6 (PMC6098152; doi:10.1038/s41467-018-05862-6)
Supplement: Supplementary file 1 — Supplementary Information [file 41467_2018_5862_MOESM1_ESM.pdf]

Supplementary information

mTOR-dependent phosphorylation controls TFEB nuclear export

Napolitano et al.

Supplementary Fig. 1

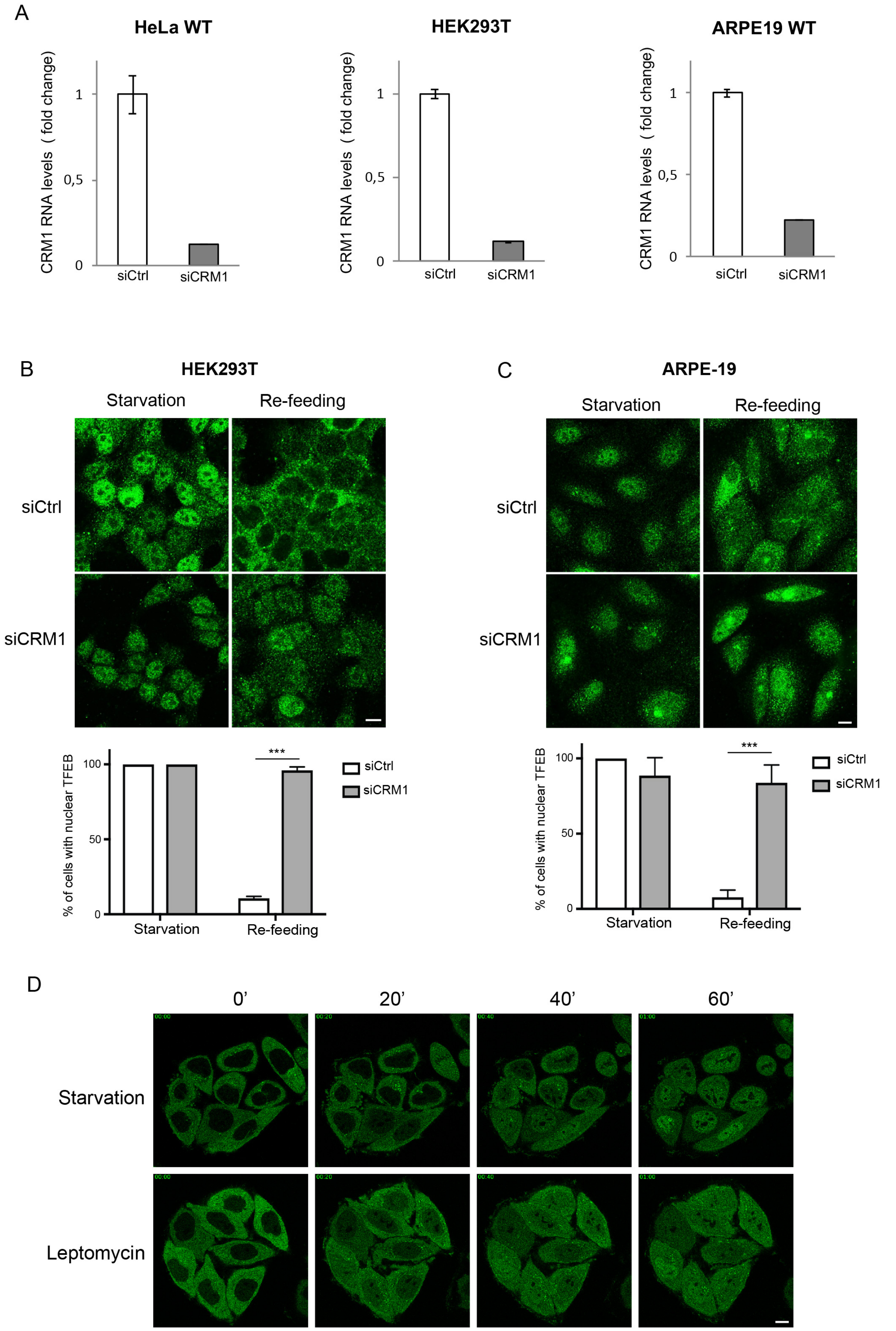

Supplementary Fig. 1. TFEB undergoes CRM1-dependent nuclear export. **A.** Quantitative RT-PCR showing CRM1 knockdown efficiency in HeLa, HEK293T and ARPE 19 cells. **B.** HEK293T cells transfected with siRNA directed against CRM1 (siCRM1) or with control siRNA (siCtrl) were either starved for 60 min (starvation), or starved and re-stimulated with amino acids for 30 min and analysed by confocal microscopy. Cells were analysed to calculate the percentage of cells showing nuclear TFEB localization. Results are mean ± SEM. n>70 cells per condition. ***P<0.001, Two-way Anova. **C.** ARPE-19 cells were treated and analysed by confocal microscopy as in B. Cells were analysed to calculate the percentage of cells showing nuclear TFEB localization. Results are mean ± SEM. n>30 cells per condition. ***P<0.001, Two-way Anova. **D.** Time-lapse imaging of HeLa cells stably expressing GFP-TFEB, either starved of amino acids or treated with 5nM Leptomycin B in the presence of nutrients, and imaged every 2 minutes by airyscan microscopy. Representative images at the indicated time points are shown.

Supplementary Fig. 2

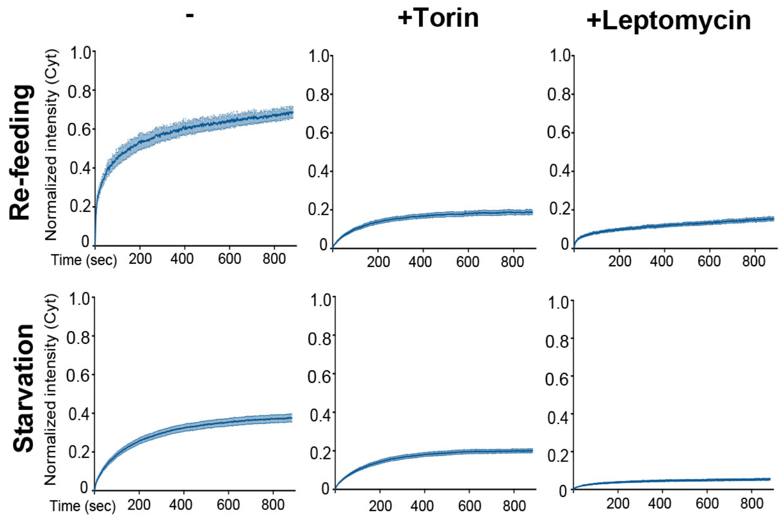

Supplementary Fig. 2. Kinetic analysis of TFEB nucleo-cytoplasmic shuttling.
Cells described in Fig. 2B and 2C were analysed and plotted for the recovery of TFEB cytosolic fluorescence using the Image J software. The plots show the normalized fluorescence recovery starting from the first frame after photobleaching for each treatment. Results are mean ± SEM. n>6 cells per condition.

Supplementary Fig. 3

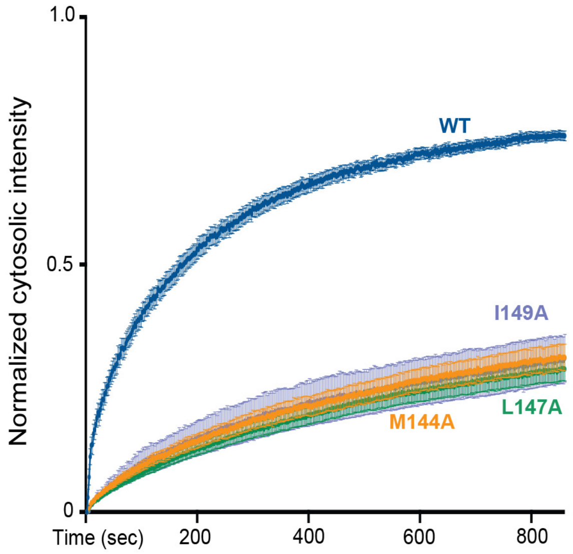

Supplementary Fig. 3. TFEB NES-mutants show impaired nuclear export kinetics. Cells described in Fig. 3E were analysed and plotted for the recovery of TFEB cytosolic fluorescence using the Image J software. The plot shows the normalized fluorescence recovery starting from the first frame after photobleaching. Results are mean ± SEM. n>12 cells per condition.

Supplementary Fig. 4

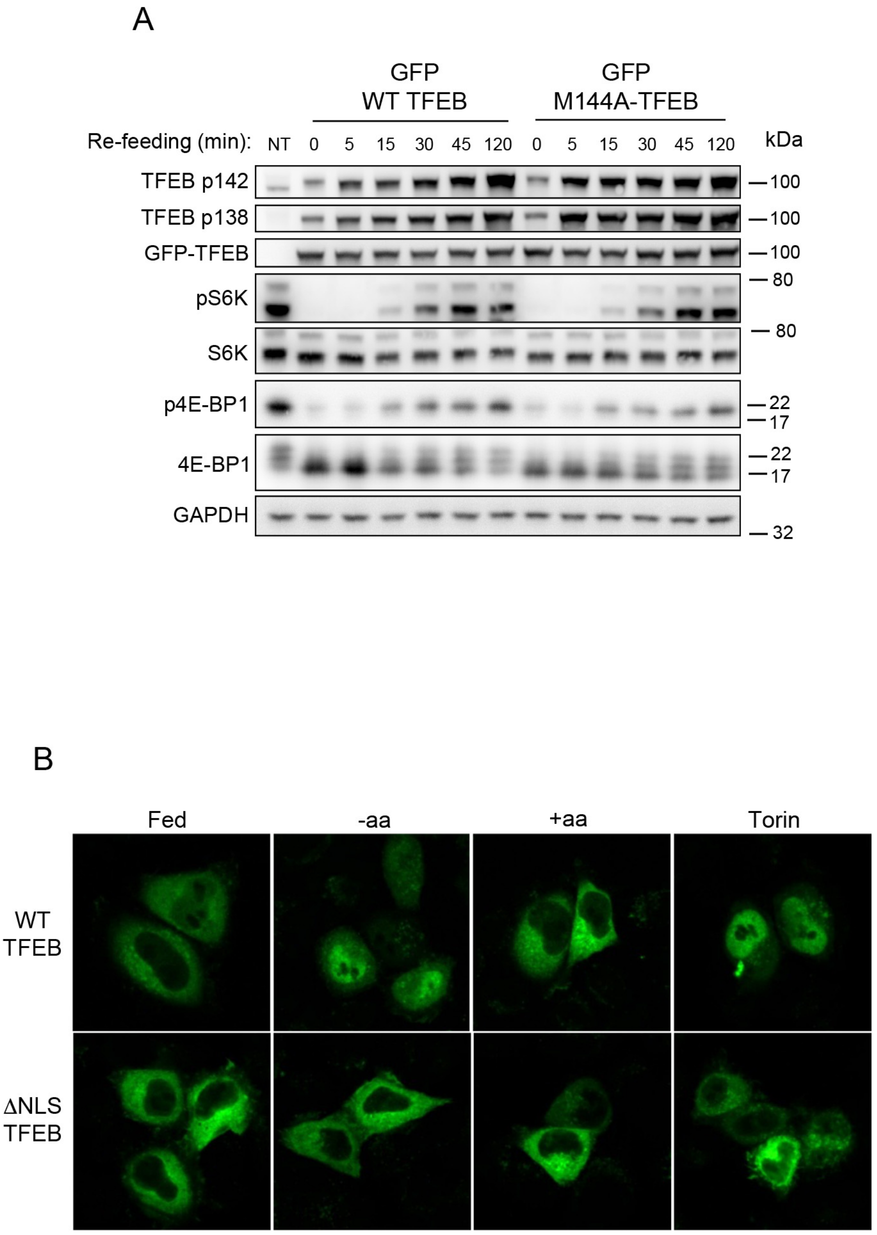

Supplementary Fig. 4. Characterization of TFEB mutants.
A. HeLa cells transfected with either wild type GFP-TFEB or M144A-GFP-TFEB were either starved for 60 min or starved and re-stimulated with amino acids the indicated time points and evaluated for TFEB phosphorylation by immunoblotting. **B.** Confocal microscopy analysis of HeLa cells transfected with either GFP-WT-TFEB or GFP-ΔNLS-TFEB and either left untreated (Fed), starved of amino acids for 60 min (-aa), or starved and re-stimulated with amino acids for 30 min in the presence or absence of Torin.

Supplementary Fig. 5

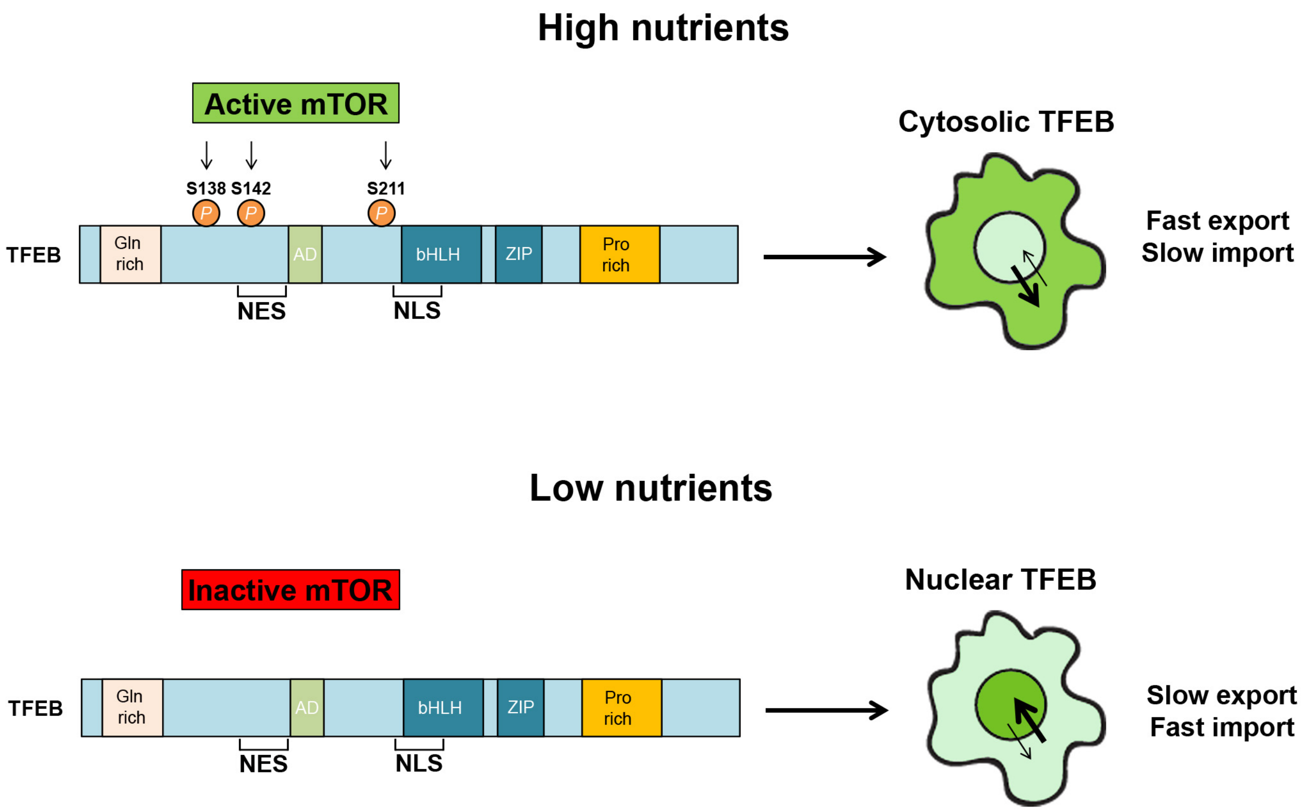

Supplementary Fig. 5
Proposed mechanism of TFEB nucleo-cytoplasmic shuttling by mTOR-dependent phosphorylation.

Supplementary Fig. 6

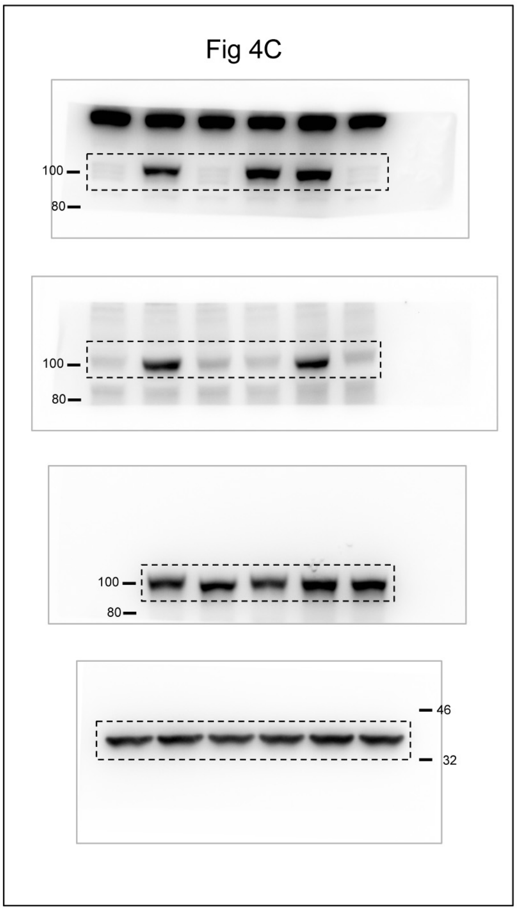

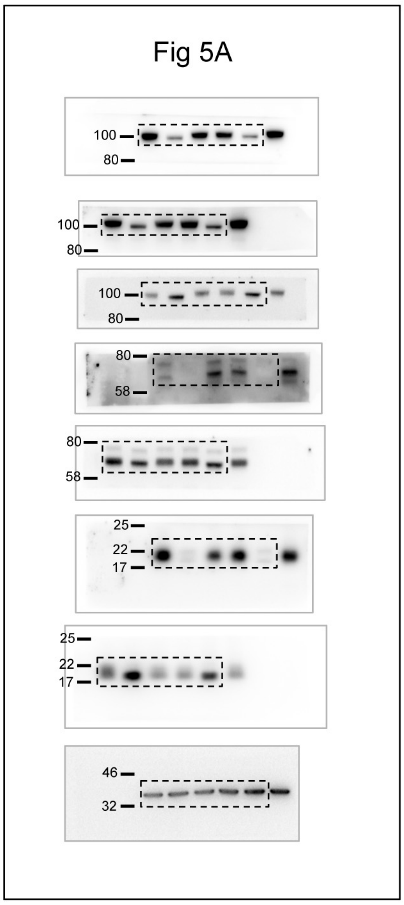

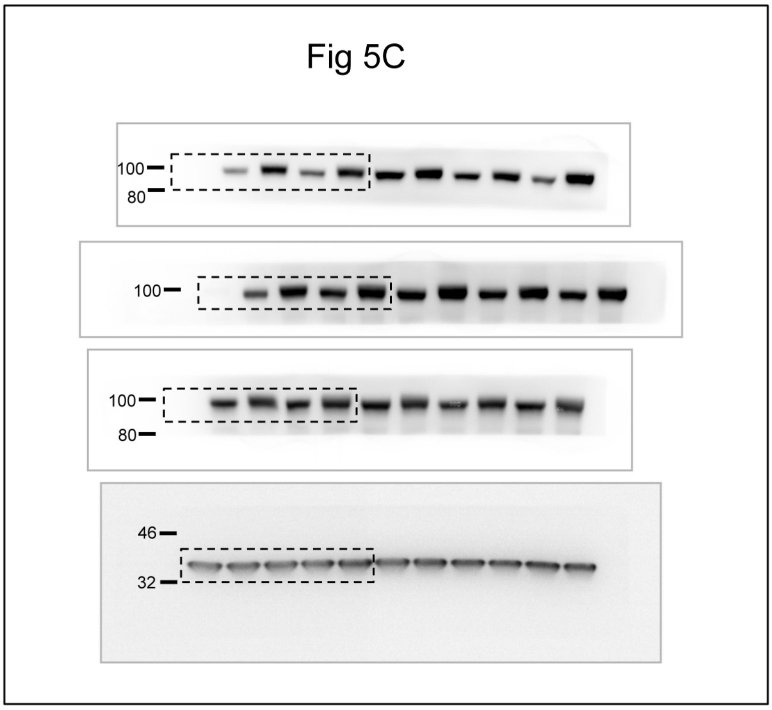

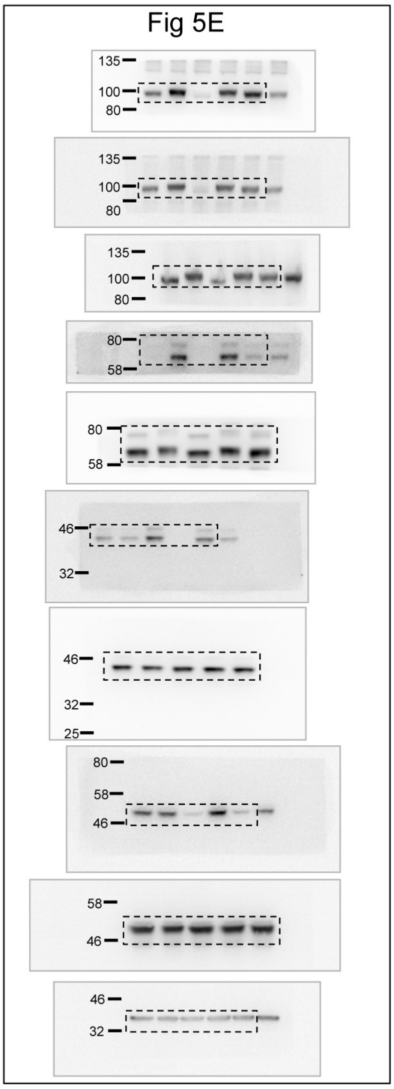

Supplementary Fig. 6
Uncropped original western blots.
